# More Progress in Eliminating Transmission of *Onchocerca volvulus* and *Wuchereria bancrofti* in the Americas: A Portent of Global Eradication

**DOI:** 10.4269/ajtmh.15-0688

**Published:** 2015-12-09

**Authors:** James W. Kazura

**Affiliations:** Center for Global Health and Diseases, School of Medicine, Case Western Reserve University, Cleveland, Ohio

Among the increasing number of “neglected tropical diseases” that have been identified to disproportionately affect the health and socioeconomic status of impoverished populations of the world, onchocerciasis, caused by the filarial nematode *Onchocerca volvulus*, and lymphatic filariasis, caused mainly by *Wuchereria bancrofti* (approximately 10% of the cases are due to the more geographically restricted filarial parasites *Brugia malayi* and *Brugia timori*), share biological and ecological features that make them feasible targets of elimination as public health problems and, ultimately, global eradication, defined as the cessation of transmission in all endemic areas in the absence of continuing public health interventions. Sustained transmission of *O. volvulus* and lymphatic filariae are dependent on the ingestion of microfilariae from the skin and blood, respectively, of infected individuals by *Simulium* blackflies and various mosquito species. Uptake of microfilariae by the arthropod vector is followed by the development of microfilariae to the infective larval stage that is necessary to continue the parasites' lifecycle in the local human population with the next cycle of blood feeding. Interventions designed to interrupt transmission of onchocerciasis and lymphatic filariasis are based on reducing the reservoir of microfilariae in the community below a threshold level that no longer sustains transmission by local vectors. Five or more years of mass drug administration (MDA), which includes various combinations of ivermectin, diethylcarbamazine, and albendazole, is the primary intervention used to reduce this reservoir, as evident in the progress of the global effort to eliminate lymphatic filariasis as a public health problem by 2020.^1^ Ancillary interventions directed at the arthropod vectors, for example, earlier attempts to reduce breeding sites of *Simulium* vectors of onchocerciasis in Africa and, more recently, distribution of insecticidal bed nets in areas of the world where malaria and lymphatic filariasis are coendemic.^2^ Challenges to the success of this strategy include the relatively high population coverage with MDA that is required to reduce the microfilarial reservoir below the threshold to stop transmission (estimated to be ≥ 65% of the total population eligible to take MDA),^3^ spatial heterogeneity of transmission and infection levels due to the proximity of human habitation to vector breeding sites, variability of transmission efficiency of various vectors,^4^,^5^ and logistic and financial constraints to the delivery of MDA and post-MDA monitoring in resource-constrained endemic countries.

Can these challenges to onchocerciasis and lymphatic filariasis elimination be overcome, and are there lessons from successful elimination efforts that can help us understand how to achieve elimination of disease and permanent cessation of transmission of *O. volvulus* and lymphatic filariae at a global level? Two articles in this issue of the *American Journal of Tropical Medicine and Hygiene* describe elimination of transmission of onchocerciasis in an area of Guatemala^6^ and bancroftian filariasis in an urban area of the Dominican Republic.^7^ The former report by Richards and others describes the process and events that led to the elimination of *O. volvulus* transmission from the central endemic zone of Guatemala, an area with the highest onchocerciasis disease burden in the Americas. Several key factors were important to elimination. First, a strong public–private collaboration, collectively referred to as the Onchocerciasis Elimination Program for the Americas,^8^ involving the Guatemala Ministry of Health working with multiple regional and international partners was critical to achieve twice yearly MDA with ivermectin that reached ≥ 85% of the eligible population over a 12-year period. This prolonged and intense MDA presumably exceeded the life span of adult female worms that produced the microfilariae required for sustaining transmission by the local *Simulium* vector. Second, a notable feature was the application of a serologic measure (Ov16 IgG4 antibody) of active infection in children born after MDA was instituted. Less than 0.1% of children under the age of 69 months were Ov16 IgG4 positive. Third, polymerase chain reaction screening of pooled *Simulium* vectors was negative, consistent with lack of an adequate reservoir of microfilariae to develop into infective larvae in the local vectors. With respect to the elimination of *W. bancrofti* transmission in an urban barrio of the Dominican Republic, Noland and others performed a transmission assessment survey (TAS) of 6- to 7-year-old children living in an area where annual MDA with diethylcarbamazine plus albedazole was given in 2004, 2005, and 2007, with population coverage ranging from 67% to 92%. In addition, school-aged children in the study area received annual to semiannual doses of albendazole for the treatment of soil-transmitted helminths before, during, and after MDA up to the time of the TAS. None of the 815 children aged 6–7 years examined in 2014 had a positive immunochromatographic test for circulating filarial antigen, indicating that the TAS criterion for stopping MDA had been reached. The instructive features of this report include the observation that, in low-transmission areas such as the Dominican Republic, fewer annual treatments with MDA may be sufficient to eliminate lymphatic filariasis transmission, particularly if additional anthelmintics, such as repeated doses of albendazole to reduce morbidity from soil-transmitted helminth infections, are given.

Can these same successes in eliminating transmission of onchocerciasis and lymphatic filariasis be realized in other endemic areas of the world? This will be difficult, especially in Africa, where the prevalence of lymphatic filariasis is higher, human population size greater, and vector abundance and transmission efficiency greater than that in the Americas. On the other hand, coendemicity of onchocerciasis and lymphatic filariasis, similarities in drugs and MDA schedule, and integration of control programs for these two filarial diseases in Africa suggest that elimination can be achieved.^9^,^10^ Lessons learned from eliminating transmission in the Americas include the importance of public–private partnerships, ownership of programs at the country and regional level, diligence to high MDA population coverage, and using scientifically rational criteria for stopping MDA and certifying transmission elimination.
